# Exploring the Benefit of 2-Methylbutyric Acid in Patients Undergoing Hemodialysis Using a Cardiovascular Proteomics Approach

**DOI:** 10.3390/nu11123033

**Published:** 2019-12-12

**Authors:** Ping-Hsun Wu, Yi-Wen Chiu, Hsin-Bai Zou, Cheng-Chih Hsu, Su-Chu Lee, Yi-Ting Lin, Yi-Chun Tsai, Mei-Chuan Kuo, Shang-Jyh Hwang

**Affiliations:** 1 Graduate Institute of Clinical Medicine, College of Medicines, Kaohsiung Medical University, Kaohsiung 80708, Taiwan; 970392@kmuh.org.tw; 2 Faculty of Medicine, College of Medicine, Kaohsiung Medical University, Kaohsiung 80708, Taiwan; lidam65@yahoo.com.tw (Y.-C.T.); mechku@kmu.edu.tw (M.-C.K.); sjhwang@kmu.edu.tw (S.-J.H.); 3 Division of Nephrology, Department of Internal Medicine, Kaohsiung Medical University Hospital, Kaohsiung 80708, Taiwan; chiuyiwen@kmu.edu.tw (Y.-W.C.); suchle5910@gmail.com (S.-C.L.); 4 Department of Medical Sciences, Uppsala University, 752 36 Uppsala, Sweden; 5 Faculty of Renal Care, College of Medicine, Kaohsiung Medical University, Kaohsiung 80708, Taiwan; 6 Department of Chemistry, National Taiwan University, Taipei 10617, Taiwan; d04223115@ntu.edu.tw (H.-B.Z.); ccrhsu@ntu.edu.tw (C.-C.H.); 7 Department of Family Medicine, Kaohsiung Medical University Hospital, Kaohsiung 80708, Taiwan; 8 Division of General Medicine, Kaohsiung Medical University Hospital, Kaohsiung Medical University, Kaohsiung 80708, Taiwan

**Keywords:** short-chain fatty acids, 2-methylbutyric acid, target proteomics, bone morphogenetic protein 6, hemodialysis, end-stage renal disease

## Abstract

Short-chain fatty acids (SCFAs) can reduce pro-inflammatory parameters and oxidative stress, providing potential cardiovascular (CV) benefits. Although some evidence links SCFAs with host metabolic health via several biological mechanisms, the role of SCFA on CV disease in patients with kidney disease remains unclear. Herein, we investigate the association between a SCFA, 2-methylbutyric acid, and target CV proteomics to explore the potential pathophysiology of SCFA-related CV benefit in patients with kidney disease. Circulating 2-methylbutyric acid was quantified by high-performance liquid chromatography and 181 CV proteins by a proximity extension assay in 163 patients undergoing hemodialysis (HD). The associations between 2-methylbutyric acid and CV proteins were evaluated using linear regression analysis with age and gender, and multiple testing adjustment. The selected CV protein in the discovery phase was further confirmed in multivariable-adjusted models and evaluated by continuous scale association. The mean value of circulating 2-methylbutyric acid was 0.22 ± 0.02 µM, which was negatively associated with bone morphogenetic protein 6 (BMP-6) according to the false discovery rate (FDR) multiple testing adjustment method. The 2-methylbutyric acid level remained negatively associated with BMP-6 (β coefficient −1.00, 95% confidence interval −1.45 to −0.55, *p* < 0.001) after controlling for other CV risk factors in multivariable models. The cubic spline curve demonstrated a linear relationship. In conclusion, circulating 2-methylbutyric acid level was negatively associated with BMP-6, suggesting that this pathway maybe involved in vascular health in patients undergoing HD. However, further in vitro work is still needed to validate the translation of the mechanistic pathways.

## 1. Introduction

Chronic kidney disease (CKD) is associated with high morbidity and mortality. Indeed, patients undergoing hemodialysis (HD) have a 10–50-fold higher risk of mortality than the age-matched general population [1]. Cardiovascular (CV) disease is an important cause of death in CKD and end-stage renal disease (ESRD) patients [2,3]. Currently, CKD is treated as an independent risk factor for CV disease and considered as a coronary disease risk equivalent [4]. The accumulation of uremic toxins correlates with systemic inflammation, protein wasting and accelerated CV complications in HD patients [5]. By contrast, short-chain fatty acids (SCFAs), which are fermented by colonic or distal small intestinal bacteria from indigestible polysaccharides or proteins, have been shown to reduce pro-inflammatory reactions, oxidative stress, insulin resistance, and improve iron bioavailability in HD patients [6]. In addition, plasma SCFA levels are also associated with CV outcomes in CKD patients [7]. 

SCFAs are fatty acids with a carbon number of not greater than 6, including three major SCFAs, acetic acid, propionic acid, butyric acid, and two less abundant SCFAs, valeric acid and caproic acid [8], in straight conformations, whereas branched SCFAs (isobutyric acid, 2-methylbutyric acid, and isovaleric acid) are mainly derived from proteins and amino acids [9]. Considering the overall hydrophobicity and low molecular weights of the SCFAs, acetic acid, propionic acid, and butyric acid can be easily absorbed via nonionic diffusion across the apical membrane of colonocytes [10]. Furthermore, SCFA has histone deacetylase (HDAC) inhibitory activity, which can alter gene expression by inhibiting histone deacetylases and altering histone acetylation [11,12]. The anti-inflammatory effect of SCFA mediated by the inhibition of HDAC could prevent the infiltration of immune cells from the bloodstream in the adipose tissue [13]. SCFA can also improve insulin sensitivity [14] and protects against sarcopenia [15]. In addition, SCFA exhibits an atherosclerosis protective effect, arresting vascular smooth muscle cell proliferation, which suggests a potential role for SCFA in CV disease prevention [16]. The mechanisms of action of SCFA are multiple, but many of these are related to its regulatory effects on gene expression involved in CV risk factors. 

Fecal analysis has demonstrated that patients with CKD or HD have lower levels of gut bacteria predicted to generate SCFA (produced by bacteria of the *Firmicutes* phylum members-*Butyricicoccus, Oscillibacter, and Dialister*) [17,18,19]. In addition, there is a negative correlation between SCFA-producing bacteria (*Roseburia spp*., *Faecalibacterium prausnitzii*) and inflammation in CKD patients compared to healthy controls [19]. Despite the evidence suggesting that SCFA has CV protection effects in the general population, the impact of this important SCFA on HD patients has not been fully elucidated. Specifically, the role of gut-produced SCFA in CV disease remains unclear in CKD or HD patients. Thus, we investigated the association between SCFA and CV-specific proteins to explore the potential pathophysiology in the clinical setting.

## 2. Materials and Methods

### 2.1. Subjects

Patients aged above 30 years old with regular HD who signed the informed consent form in Kaohsiung Medical University Hospital (KMUH) were recruited from August 2016 through January 2017. All patients received at least 90 days regular HD three times per week at a rate of 250 to 300 ml/min with high-efficiency dialyzers, 500 ml/min on dialysate flow, lasting 3.5–4 h per session, and received adequate dialysis (Kt/V > 1.2). The study protocol was approved by the Institutional Review Board of Kaohsiung Medical University Hospital (KMUHIRB-E(I)-20160095 and KMUHIRB-E(I)-20180139).

### 2.2. Comorbidity and Biochemical Measurements

The disease course, comorbidities, and drug prescription were obtained from electronic health care system records. The baseline characteristics included age, sex, the primary cause of kidney failure (hypertension, diabetes, glomerulonephritis, and others), time on dialysis, dialysis access (fistula vs. graft), comorbidities, medication treatment, and biochemistry profile for all participants. Biochemical data from blood samples were obtained after overnight fasting for each participant through the arteriovenous fistula or graft. The blood sample was transported to the central freezer and stored at −80 °C. The definition of hypertension was based on a blood pressure of 140/90 mmHg or higher or taking anti-hypertensive drugs. Patients were defined as having diabetes mellitus based on a HbA1C of 6.5% or higher or taking antidiabetic drugs.

### 2.3. High-Performance Liquid Chromatography for Short-Chain Fatty Acid Profiling

Serum SCFA analysis was via high-performance liquid chromatography (HPLC). The derivatization method was followed with the previously reported protocol and with minimal laboratory modification for optimal efficiency [20,21]. In brief, each aliquot of 100 µL serum sample was spiked with a solution of 10 µL of ethanol containing 1 nmol of 2-ethylbutyric acid (Sigma-Aldrich, St. Louis, MO, USA) as the internal standard. The serum samples were treated according to the derivatization procedure with acidic 2-nitrophenylhydrazine hydrochloride (2-NPH·HCl). The resulting mixture of hydrazides was neutralized by the addition of 4 mL of 0.033 M (mol/L) phosphate buffer (pH 6.4) with 0.5 M hydrochloric acid (3.8:0.4, *v/v*). The SCFA hydrazides were extracted with 4 mL of diethyl ether. The ether layer was washed with 3 mL of 0.033 M phosphate buffer (pH 6.4) and evaporated with a stream of nitrogen at room temperature. The residue was resuspended in 50 µL of methanol, and an aliquot (20 µL) was removed for HPLC analysis. HPLC was performed using an Agilent 1260 series HPLC (Santa Clara, CA, USA) and a C-18 reverse phase column (J’sphere ODS-M80, 250 x 4.6 mmI.D. S-4 μM, 8 nm, YMC, Kyoto, Japan). The mobile phase A was water, mobile phase B was acetonitrile-methanol (30:16, *v/v*), where 0.1% trifluoroacetic acid (TFA; Special Grade, Wako Pure Chemical Industries, Osaka, Japan) was added to both solvents in mobile phase A and B. The column temperature was set at 45 °C, the flow rate was 1 mL/min, and detector wavelength was set at 400 nm. In total, 9 commercial standard compounds of SCFAs, including lactic acid, acetic acid, propionic acid, isobutyric acid, butyric acid, 2-methylbutyric acid, isovaleric acid, valeric acid, isocaproic acid, were first obtained as reference chromatogram (Supplementary Figure S1). The standards were treated with the same derivatization procedure as that for serum samples before HPLC analysis. After that, we compared the retention time of the derivatized standards with each observed SCFA in serum samples. The SCFA was identified according to their retention time as compared with the standards (Supplementary Figure S2). This method is routinely used in SCFA analysis, and the details of the procedures have been described in a previous report [20,21]. Sample values below the lower limit of detection ((LOD) > 15%) were excluded as quality control.

### 2.4. Proteomic Profiling

The Proseek Multiplex proximity extension assay (Olink Bioscience, Uppsala, Sweden) was used to assess proteins from HD serum samples. The highly specific assay can measure 184 CV-specific proteins (Supplementary Table S1) simultaneously using two antibodies per protein which were pairwise bound to each protein. When both antiboties are bound to the surface of target protein, a polymerase chain reaction (PCR) sequence from attached oligonucleotide strands will be create. To determine the lower detection limit and normalize the levels for each subject, two incubations, one extension, and one detection control were used. The resulting relative values were log_2_-transformed for subsequent analysis, and each protein level was normalized per plate by setting the mean to zero and standard deviation to one within each plate and storage [22]. The mean intra-assay and inter-assay coefficients of variation were 4% and 10%, respectively. Quantitative PCR quantification cycle (Cq) values determined protein expression (NPX) values, where higher Cq corresponds to lower protein abundance. Cq values (log_2_ scale) were corrected for technical variation by an inter-plate control, and lower LOD were determined by a negative control. Values below the LOD were imputed as LOD/2 and normalized per plate. Quality control included the removal of proteins with >15% samples below the LOD and subjects which could not pass internal quality control were excluded.

### 2.5. Statistical Analysis

The demographic and baseline characteristics are presented as the mean ± SD for continuous variables and percentages for ordinal or nominal variables. For the first analysis phase, the associations between 2-methylbutyric acid with the 184 CV proteins (each in a separate model) were investigated using linear regression models adjusting multiple testing models by a false discovery rate (FDR) < 5%. The FDR was calculated based on the original version of Benjamini and Hochberg (1995) [23]. Moreover, the proteins were ranked via protein-bound uremic toxins by an ascending *p*-value, with confidence intervals around the ranks using bootstrapping. In the second phase, multivariable-adjusted models were used according to a causal diagram by a directed acyclic graph (DAG) from DAGitty, version 2.2, software (Supplementary Figure S3), and the models contained the covariates age, sex, hemodialysis duration, cause of ESRD, arteriovenous shunt type, diabetes mellitus, hypertension, dyslipidemia, antiplatelet/warfarin, anti-hypertensive drugs, diabetic treatment drugs, calcium, phosphate, high sensitivity C-reactive protein (hsCRP), and total Kt/V. Proteins found to be statistically significant were further investigated using linear regression splines. Stata (version 15, College Station, TX, USA) was used for all statistical methods. Results were reported as a beta coefficient (β), with a 95% confidence interval (CI), and two-tailed *p* < 0.05 was considered statistically significant in the second phase analysis.

## 3. Results

**Figure 1:**
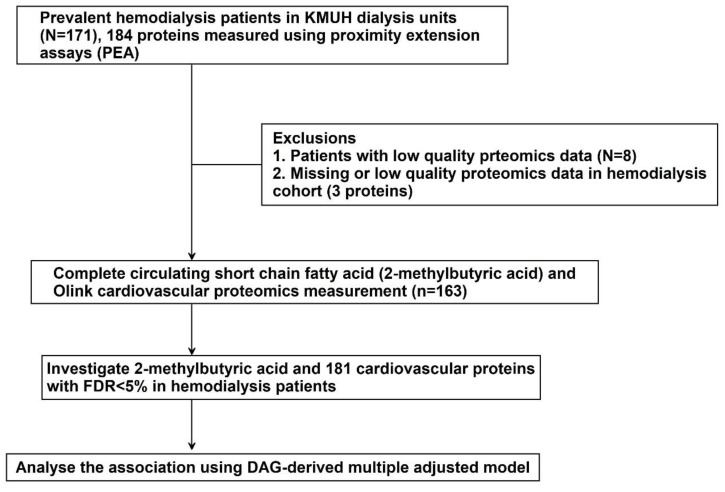
Study design.

In total, 171 HD patients were enrolled for the measurement of nine SCFAs by HPLC and 184 CV-specific proteins by proximity extension assays. After quality control of SCFA data, lactic acid and 2-methylbutyric acid were the major SCFAs detected in blood samples of HD patients. However, exogenous lactic acid formed by lactic acid bacteria enters the blood compartment and is mixed with endogenous lactic acid released by tissues and organs. Thus, we mainly investigate 2-methylbutyric acid because our study aim was focused on gut-produced SCFAs. In addition, subjects with low-quality proteomics data (*n* = 8) were excluded. Three proteins were excluded because of low-quality proteomics data. Finally, the association between 2-methylbutyric acid and 181 CV cascade proteins was analyzed in 163 HD patients (Figure 1). 

### 3.1. Demographic and Clinical Characteristics

The characteristics of the included patients are listed in Table 1. The mean age of the HD patients was 58.7 ± 12.2, and 53.4% were male, 82.2% had hypertension, and 36.8% had diabetes. The cause of ESRD was glomerulonephritis (33.7%), followed by diabetes (26.4%). The mean years of dialysis vintage were 6.35 ± 5.98 and the arteriovenous fistula was more popular in these patients (87.1%). The mean serum level of ionized calcium was 4.6 ± 0.41 mmole/L, 4.66 ± 1.05 mmol/L for phosphate, and 4.29 ± 5.38 mg/dL for hsCRP. The mean serum level of 2-methylbutyric acid was 0.22 ± 0.02 µM (Table 1). The mean NPX value of target CV proteins is demonstrated in Supplementary Table S1.

### 3.2. Discovery Phase

**Figure 2:**
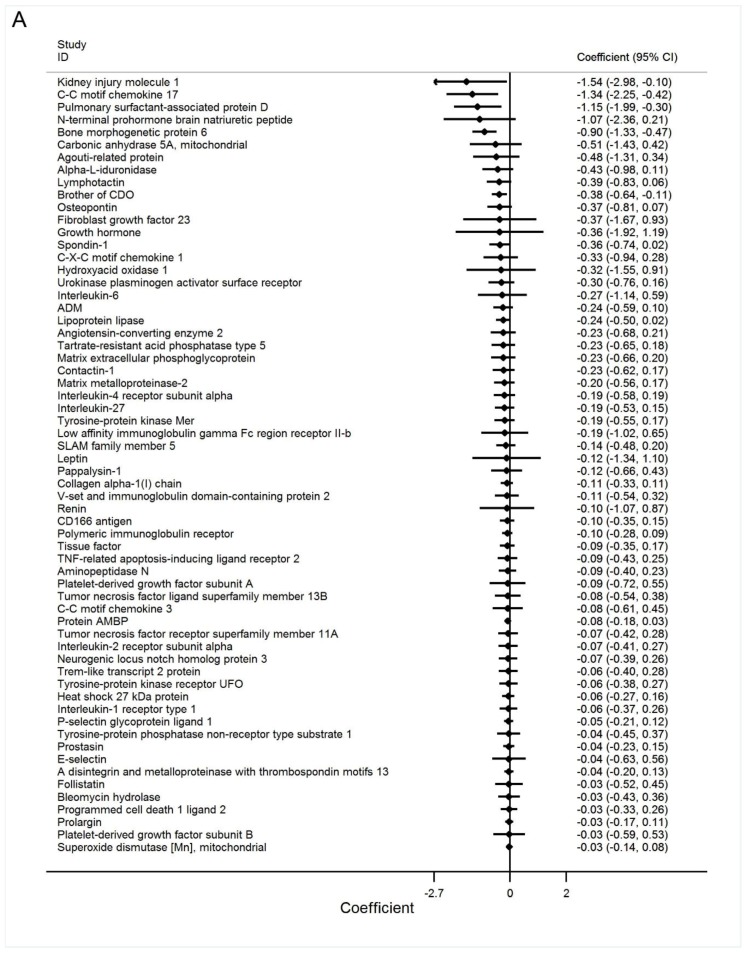

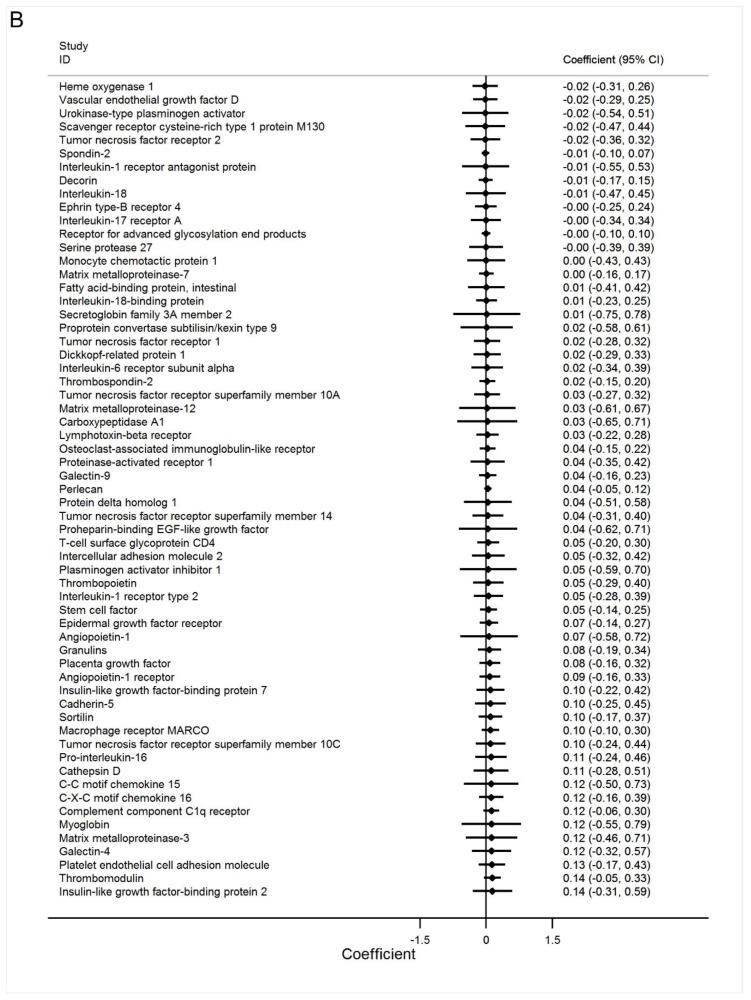

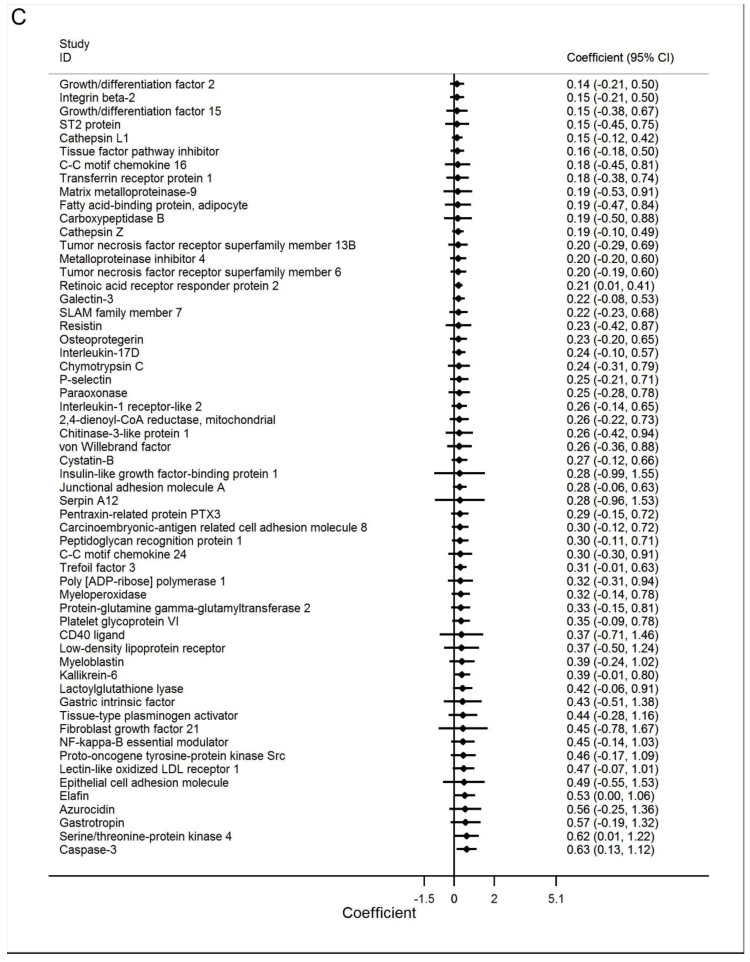
The association between 2-methylbutyric acid and 181 cardiovascular proteins in linear regression models with age and sex adjustment. (**A**) protein 1–62 (**B**) protein 63–123 (**C**) protein 124–181.

**Figure 3:**
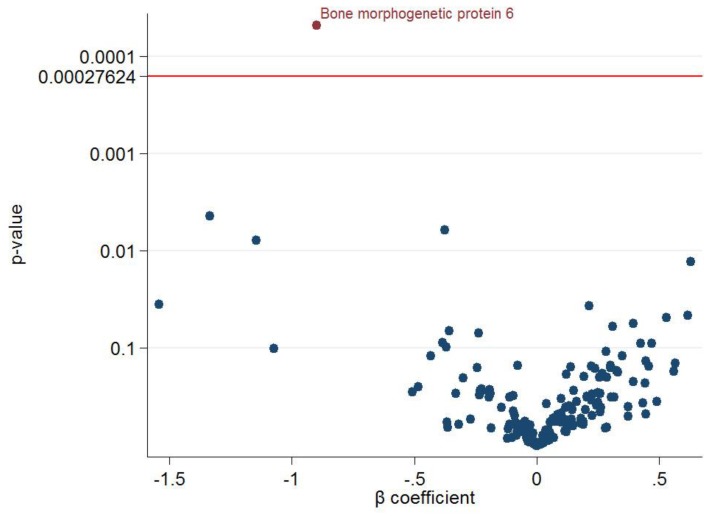
Volcano plot of the *p*-value and β coefficient for the association between 2-methylbutyric acid and 181 cardiovascular protein biomarkers with a false discovery rate <5% multiple testing control.

When relating the 2-methylbutyric acid to 181 CV-specific proteins one by one in HD patients controlling age and sex, the circulating 2-methylbutyric acid level was negatively associated with five proteins (kidney injury molecule 1, C-C motif chemokine 17, pulmonary surfactant-associated protein D, N-terminal prohormone brain natriuretic peptide, and bone morphogenetic protein 6 [BMP-6]) and positively associated with two proteins (serine/threonine-protein kinase 4 and caspase-3) (Figure 2A–C and Supplementary Table S2). Using an FDR of 5% (corresponding to *p* = 0.00027624), 2-methylbutyric acid remained significantly associated with BMP-6 (Figure 3). The ranking of the associations (with 95% bootstrap-obtained CIs) of 2-methylbutyric acid with all CV proteins is graphically presented. The protein BMP-6 was the top hit related to 2-methylbutyric acid, with wide confidence intervals of the ranking as expected (Supplementary Figure S4).

### 3.3. Best Estimates Phase

**Figure 4:**
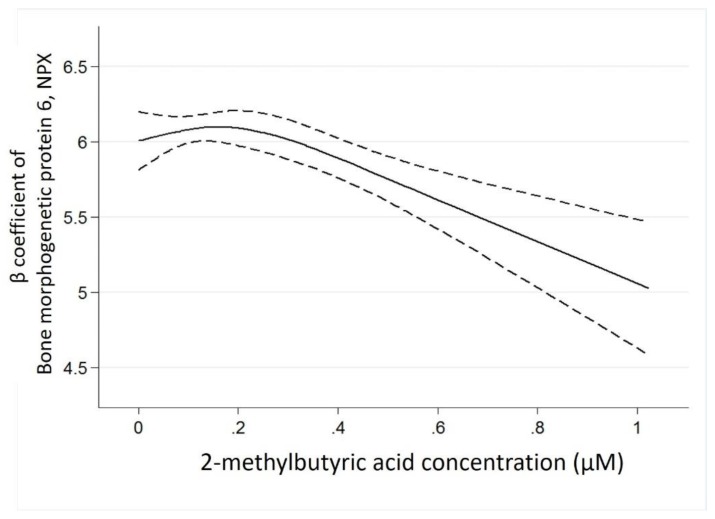
The cubic spline curve (with 3 knots) illustrating the associations of 2-methylbutyric acid level and selected circulating cardiovascular protein biomarker determined protein expression (NPX) units.

Investigating associations between 2-methylbutyric acid and BMP-6 in a multivariable-adjusted linear regression model, the negative association (β coefficient −1.00, 95% CI −1.45 to −0.55, *p* < 0.001) persisted after adjusting for baseline age, sex, hemodialysis vintage, cause of ESRD, arteriovenous shunt type, comorbidities (diabetes mellitus, hypertension, and dyslipidemia), medications (antiplatelet/warfarin drugs, anti-hypertensive drugs, and diabetic treatment drugs), and clinical laboratory data (serum ionized calcium level, phosphate level, hsCRP level, and Kt/V) (Table 2). Cubic spline analysis demonstrated a chiefly linear association of 2-methylbutyric acid and BMP-6 (Figure 4).

## 4. Discussion

### 4.1. Principal Observations

In this study, we investigated associations between 2-methylbutyric acid with a large number of circulating CV-specific proteins in an HD cohort. Accounting for multiple testing, and in multivariable-adjusted linear regression models, circulating 2-methylbutyric acid levels were negatively associated with BMP-6. The 2-methylbutyric acid, known as a branched-chain SCFA, is produced mainly through the fermentation of protein-derived branched chain amino acids [24,25]. In comparison with straight-chain SCFAs, the absorption and metabolism of colonic branched-chain SCFAs has been little investigated. In humans, the SCFA content in the colon region represents at least 100 mM, while its concentration ranges from 0.1 to 10 mM in the bloodstream [26]. Although decreased numbers of SCFA-producing gut microbiota in CKD or HD patients were investigated [17,18], the concentration or uptake of SCFA in this population has not extensively measured. This study also provided fundamental information about the 2-methylbutyric acid level in HD patients. To our knowledge, it is the first study to test the association of 2-methylbutyric acid with CV protein association to explore the potential CV effect in clinical setting. Moreover, we believe that the knowledge gained here can be generalized, and thereby impact the potential CV benefit of SCFA in patients with kidney disease.

### 4.2. The Cardiovascular Benefit of Short-Chain Fatty Acids

SCFAs are generated by the degradation and fermentation of indigestible carbohydrates by gut microbiota. Approximately 90% of the SCFAs formed are absorbed in the colon then transported through the hepatic vein to the liver [27] but only a small amount reaches the blood circulation and is used as energy by the host [28]. SCFAs act on the G protein-coupled receptor (GPCR) expressed on the plasma membrane of target cells distributed widely in mammals [29]. Various health-promoting effects have been ascribed to specific SCFAs on gut barrier integrity, inflammatory and immune response, as well as the modulation of glucose and lipid metabolism [3]. 

SCFAs exert anti-inflammatory actions both in intestinal epithelial cells [30] and in the CV system [31]. SCFAs could prevent inflammation through suppressing pro-inflammatory cytokines, such as interleukin (IL)-6, IL-12, IL-1β, tumor necrosis factor-α, and nitric oxide [32,33], and increasing the anti-inflammatory cytokine IL-10 [34,35] via the stimulation of G protein receptors (GPRs) [36,37] or the inhibition of HDAC [38]. Moreover, SCFAs induce IL-10-expressing regulatory T cells to reduce inflammation [39,40]. SCFA supplementation to HD patients can alleviate pro-inflammatory parameters with a decline of CRP, IL-2, IL-6, IL-17, and interferon-ƴ and increase in anti-inflammatory IL-10 [6].

Thus, heart-healthy diets are high in dietary fiber, which largely comes from vegetables and fruits, and may lead to the production of protective SCFAs [8]. In ESRD patients, reduced dietary fiber intake was associated with a reduction in SCFA-forming bacteria [17], and colonic CKD pathology ameliorated after improving dietary management [41]. However, a high fiber diet usually contains high levels of potassium and phosphorus, which can be associated with increased mortality risk in HD patients with poor clearance [42,43]. Despite the indirect positive effects of SCFAs on CKD/ESRD patients, further study is required.

### 4.3. The Potential Mechanism to Link 2-Methylbutyric Acid and Bone Morphogenetic Protein 6

SCFA can activate GPR41, GPR43, and GPR109A [26,44]. GPR stimulation can activate intracellular signaling cascades, involving nuclear transcription, enzyme activation, and ion transport on cell membrane [26]. Therefore, SCFAs may alter vascular physiology [45], modulate adipocyte metabolism [46], and enhance the immune response [47], leading to the resultant increase in CV disease witnessed in the CKD population.

Members of the bone morphogenetic protein (BMP) superfamily were reported to be involved in atherosclerotic lesions and implicated in the pathogenesis of CV disease [48,49]. BMPs mediate vascular calcification [50] and loss of matrix Gla protein (an endogenous BMP inhibitor) causes extensive calcification of elastic and muscular arteries [51], suggesting the essential role of BMP activity regulation for maintaining a normal vessel media. BMPs, along with the Wnt family of glycoproteins, are the most important anabolic factors in bone formation [49]. Vascular calcific lesions associated with atherosclerosis, diabetes, and CKD are known to be enriched in BMP ligands and cells with the phenotypic profile of osteoblasts, whose differentiation is known to be coordinated by BMPs [52]. Vascular calcification in CKD was regulated in a similar process as bone formation, so it is intuitive to consider the BMPs in the pathogenesis of vascular calcification [49]. BMP-6 was also found localized to areas of vascular calcification as expected [53]. Interestingly, our finding of a negative association between 2-methylbutyric acid and BMP-6 may link to the potential effect on vascular calcification. SCFA, as an HDAC inhibitor [11], is a key epigenetic regulator for kidney injury and downregulation of Klotho expression [54,55]. However, the pathophysiologic background of these findings remains to be elucidated. Study in this area will provide insight into whether increasing circulating SCFAs provide any direct clinical benefit, which could ultimately result in a new CV therapeutic approach for people with HD.

### 4.4. Strengths and Limitations

This is the first study to investigate possible biological pathways using a proximity extension assay-based modern technology proteomics chip that allows for rapid high-throughput analysis of high sensitivity and specificity at the same time. Nonetheless, this study has limitations. First, a causal inference could not be drawn because of the cross-sectional study design. Besides, circulating SCFA or proteomics levels were not evaluated in non-HD controls in this study. The association still requires further work to validate the translation of the mechanistic pathways suggested in vitro. Second, the participants in the present study were hemodialysis patients, so the generalizability to patients with peritoneal dialysis or CKD should be caution. In addition, these results should be applied to other races with caution. Third, physical activity and diet habits were not included in our study, which could influence the level of SCFA or proteins. Last, this study only included CV-specific proteins. An untargeted proteomics study design could possibly identify other associated proteins and underlying mechanisms. Thus, the proposed mechanisms through which proteins may be pathophysiologically related to SCFA are hypothesis-generating.

## 5. Conclusions

We found a negative association between 2-methylbutyric acid and BMP-6 that may contribute to the knowledge on the influence of SCFAs on CV disease. Vascular calcification (BMPs) may be a possible mechanism of SCFA-associated potential CV benefit in HD patients. 

## Figures and Tables

**Table 1:** Baseline characteristics of hemodialysis participants.

	*N* = 163
Age (years)	58.7 ± 12.2
Male	87 (53.4%)
Hemodialysis vintage (years)	6.35 ± 5.98
Cause of end-stage renal disease (ESRD)	
Hypertension	25 (15.3%)
Diabetes Mellitus	43 (26.4%)
Glomerulonephritis	55 (33.7%)
Others *	40 (24.5%)
Arteriovenous shunt	
Arteriovenous fistula	142 (87.1%)
Arteriovenous graft	21 (12.9%)
Comorbidities	
Diabetes mellitus	60 (36.8%)
Hypertension	134 (82.2%)
Dyslipidemia	52 (31.9%)
Medications	
Antiplatelets/Warfarin	50 (30.7%)
Anti-hypertensive drugs	78 (47.9%)
Diabetic treatment drugs	47 (28.8%)
Laboratory data	
Ionized calcium, mmole/L	4.6 ± 0.41
Phosphate, mmol/L	4.66 ± 1.05
High sensitivity C-reactive protein, mg/L	4.29 ± 5.38
Total Kt/V	1.55 ± 0.22
Short-chain fatty acid	
2-Methylbutyric acid, µM	0.22 ± 0.02

* Other causes of end-stage renal disease included polycystic kidney disease, tumor, systemic lupus erythematosus, gout, and interstitial nephritis.

**Table 2:** Associations between circulating 2-methylbutyric acid levels and selected cardiovascular protein biomarkers in the multivariate linear regression model.

	β Coefficient (95% CI)	*p*–Value
Bone morphogenetic protein 6	−1.00 (−1.45 to −0.55)	<0.001

Multivariate linear regression model adjusting for age, sex, hemodialysis duration, systolic blood pressure/diastolic blood pressure, cause of end-stage renal disease, arteriovenous shunt type, comorbidities (diabetes mellitus, hypertension, and dyslipidemia), medications (antiplatelet/warfarin, anti-hypertensive drugs, diabetic treatment drugs), and laboratory data (calcium, phosphate, high sensitivity C-reactive protein, and Kt/V).

## Supplementary Materials

The following are available online at https://www.mdpi.com/2072-6643/11/12/3033/s1, Table S1. List of 184 proteins measured by proximity extension assay, Table S2. The β coefficient and 95% CI in linear regression models with age and sex adjustment between 2-methylbutyric acid and 181 cardiovascular proteins, Figure S1. Chromatogram obtained using UV detection (λ = 400 nm) of nine standard SCFA mixture reacted with 2-nitrophenylhydrazides, Figure S2. Chromatogram of derivatized SCFA in human serum sample, Figure S3. Proposed causal diagram for the association between short chain fatty acid and cardiovascular protein biomarkers, Figure S4. Ranking proteins by *p*-value with bootstrapped confidence intervals around the ranks related to 2-methylbutyric acid.
