# Supplementary material for: Exploring the Benefit of 2-Methylbutyric Acid in Patients Undergoing Hemodialysis Using a Cardiovascular Proteomics Approach
Source: Nutrients. 2019 Dec 12;11(12):3033. doi: 10.3390/nu11123033 (PMC6950398; doi:10.3390/nu11123033)
Supplement: Supplementary file 1 [file nutrients-11-03033-s001.docx]

**Supplementary Table S1.** List of 184 proteins measured by proximity extension assay. We exclude 3 proteins that failed quality control metrics from the analysis and present mean NPX value and standard deviation in 163 hemodialysis patients

| UniProt ID | Abbreviation | Protein name | Excluded | Mean NPX value ± SD |
| --- | --- | --- | --- | --- |
| Q9BYF1 | ACE2 | Angiotensin-converting enzyme 2 |  | 4.318 ± 0.59 |
| Q76LX8 | ADAM-TS13 | A disintegrin and metalloproteinase with thrombospondin motifs 13 |  | 7.381 ± 0.19 |
| P35318 | ADM | ADM |  | 8.625 ± 0.373 |
| O00253 | AGRP | Agouti-related protein |  | 7.537 ± 0.871 |
| Q13740 | ALCAM | Aminopeptidase N |  | 8.227 ± 0.298 |
| P02760 | AMBP | Protein AMBP |  | 8.688 ± 0.116 |
| Q15389 | ANG-1 | Angiopoietin-1 |  | 10.013 ± 0.726 |
| P15144 | AP-N | Azurocidin |  | 5.493 ± 0.347 |
| P30530 | AXL | CD166 antigen |  | 10.704 ± 0.34 |
| P20160 | AZU1 | Tyrosine-protein kinase receptor UFO |  | 4.627 ± 0.957 |
| Q13867 | BLM hydrolase | Bleomycin hydrolase |  | 2.522 ± 0.421 |
| P22004 | BMP-6 | Bone morphogenetic protein 6 |  | 6.023 ± 0.487 |
| P16860 | BNP | Natriuretic peptides B | Y |  |
| Q9BWV1 | BOC | Brother of CDO |  | 5.341 ± 0.286 |
| P35218 | CA5A | Carbonic anhydrase 5A, mitochondrial |  | 4.879 ± 1.015 |
| P42574 | CASP-3 | Caspase-3 |  | 4.827 ± 0.553 |
| Q16663 | CCL15 | C-C motif chemokine 15 |  | 10.291 ± 0.673 |
| O15467 | CCL16 | C-C motif chemokine 16 |  | 8.65 ± 0.691 |
| Q92583 | CCL17 | C-C motif chemokine 17 |  | 10.157 ± 1.072 |
| O00175 | CCL24 | C-C motif chemokine 24 |  | 5.587 ± 0.648 |
| P10147 | CCL3 | C-C motif chemokine 3 |  | 8.998 ± 0.598 |
| Q86VB7 | CD163 | Scavenger receptor cysteine-rich type 1 protein M130 |  | 8.827 ± 0.505 |
| P01730 | CD4 | T-cell surface glycoprotein CD4 |  | 6.895 ± 0.284 |
| P29965 | CD40-L | CD40 ligand |  | 8.742 ± 1.196 |
| Q9UIB8 | CD84 | SLAM family member 5 |  | 6.422 ± 0.374 |
| Q9NPY3 | CD93 | Complement component C1q receptor |  | 12.601 ± 0.208 |
| P33151 | CDH5 | Cadherin-5 |  | 5.566 ± 0.395 |
| P31997 | CEACAM8 | Carcinoembryonic antigenrelated cell adhesion molecule 8 |  | 7.882 ± 0.483 |
| P36222 | CHI3L1 | Chitinase-3-like protein 1 |  | 6.838 ± 0.799 |
| Q13231 | CHIT1 | Chitotriosidase-1 | Y |  |
| Q12860 | CNTN1 | Contactin-1 |  | 5.206 ± 0.439 |
| P02452 | COL1A1 | Collagen alpha-1(I) chain |  | 4.102 ± 0.24 |
| P15085 | CPA1 | Carboxypeptidase A1 |  | 8.288 ± 0.733 |
| P15086 | CPB1 | Carboxypeptidase B |  | 7.882 ± 0.745 |
| P04080 | CSTB | Cystatin-B |  | 8.605 ± 0.446 |
| Q99895 | CTRC | Chymotrypsin C |  | 11.799 ± 0.601 |
| P07339 | CTSD | Cathepsin D |  | 3.333 ± 0.437 |
| P07711 | CTSL1 | Cathepsin L1 |  | 8.184 ± 0.288 |
| Q9UBR2 | CTSZ | Cathepsin Z |  | 7.233 ± 0.322 |
| P09341 | CXCL1 | C-X-C motif chemokine 1 |  | 10.151 ± 0.674 |
| Q9H2A7 | CXCL16 | C-X-C motif chemokine 16 |  | 6.616 ± 0.321 |
| P07585 | DCN | Decorin |  | 6.424 ± 0.185 |
| Q16698 | DECR1 | 2,4-dienoyl-CoA reductase, mitochondrial |  | 4.11 ± 0.508 |
| O94907 | Dkk-1 | Dickkopf-related protein 1 |  | 10.721 ± 0.342 |
| P80370 | DLK-1 | Protein delta homolog 1 |  | 9.897 ± 0.625 |
| P00533 | EGFR | Epidermal growth factor receptor |  | 3.585 ± 0.238 |
| P16422 | Ep-CAM | Epithelial cell adhesion molecule |  | 7.091 ± 1.11 |
| P54760 | EPHB4 | Ephrin type-B receptor 4 |  | 8.047 ± 0.27 |
| P12104 | FABP2 | Fatty acid-binding protein, intestinal |  | 10.741 ± 0.465 |
| P15090 | FABP4 | Fatty acid-binding protein, adipocyte |  | 10.747 ± 0.804 |
| P25445 | FAS | Tumor necrosis factor receptor superfamily member 6 |  | 7.915 ± 0.408 |
| Q9NSA1 | FGF-21 | Fibroblast growth factor 21 |  | 10.026 ± 1.345 |
| Q9GZV9 | FGF-23 | Fibroblast growth factor 23 |  | 7.899 ± 1.469 |
| P19883 | FS | Follistatin |  | 12.042 ± 0.517 |
| P17931 | Gal-3 | Galectin-3 |  | 4.541 ± 0.354 |
| P56470 | Gal-4 | Galectin-4 |  | 6.318 ± 0.486 |
| O00182 | Gal-9 | Galectin-9 |  | 9.464 ± 0.225 |
| Q99988 | GDF-15 | Growth/differentiation factor 15 |  | 8.978 ± 0.603 |
| Q9UK05 | GDF-2 | Growth/differentiation factor 2 |  | 8.922 ± 0.407 |
| P01241 | GH | Growth hormone |  | 8.701 ± 1.769 |
| P27352 | GIF | Gastric intrinsic factor |  | 9.779 ± 1.016 |
| Q04760 | GLO1 | Lactoylglutathione lyase |  | 7.328 ± 0.523 |
| Q9HCN6 | GP6 | Platelet glycoprotein VI |  | 4.337 ± 0.474 |
| P28799 | GRN | Granulins |  | 7.121 ± 0.299 |
| P51161 | GT | Gastrotropin |  | 5.302 ± 0.85 |
| Q9UJM8 | HAOX1 | Hydroxy acid oxidase 1 |  | 5.379 ± 1.316 |
| Q99075 | HB-EGF | Proheparin-binding EGF-like growth factor |  | 7.038 ± 0.716 |
| P09601 | HO-1 | Heme oxygenase 1 |  | 12.05 ± 0.324 |
| Q8IYS5 | hOSCAR | Osteoclast-associated immunoglobulin-like receptor |  | 12.139 ± 0.203 |
| P04792 | HSP 27 | Heat shock 27 kDa protein |  | 9.83 ± 0.246 |
| P13598 | ICAM-2 | Intercellular adhesion molecule 2 |  | 6.899 ± 0.405 |
| P35475 | IDUA | Alpha-L-iduronidase |  | 5.034 ± 0.611 |
| P08833 | IGFBP-1 | Insulin-like growth factor-binding protein 1 |  | 7.436 ± 1.427 |
| P18065 | IGFBP-2 | Insulin-like growth factor-binding protein 2 |  | 10.166 ± 0.497 |
| Q16270 | IGFBP-7 | Insulin-like growth factor-binding protein 7 |  | 10.164 ± 0.383 |
| P31994 | IgG Fc receptor II-b | Low-affinity immunoglobulin gamma Fc region receptor II-b |  | 4.767 ± 0.941 |
| Q14005 | IL16 | Pro-interleukin-16 |  | 8.363 ± 0.38 |
| Q8TAD2 | IL-17D | Interleukin-17D |  | 4.033 ± 0.42 |
| Q96F46 | IL-17RA | Interleukin-17 receptor A |  | 5.522 ± 0.368 |
| Q14116 | IL18 | Interleukin-18 |  | 9.136 ± 0.478 |
| O95998 | IL-18BP | Interleukin-18-binding protein |  | 8.16 ± 0.256 |
| P18510 | IL-1ra | Interleukin-1 receptor antagonist protein |  | 6.293 ± 0.626 |
| Q9HB29 | IL1RL2 | Interleukin-1 receptor-like 2 |  | 5.136 ± 0.496 |
| P14778 | IL-1RT1 | Interleukin-1 receptor type 1 |  | 7.77 ± 0.34 |
| P27930 | IL-1RT2 | Interleukin-1 receptor type 2 |  | 5.669 ± 0.365 |
| Q8NEV9,Q14213 | IL-27 | Interleukin-27 |  | 7.176 ± 0.387 |
| P01589 | IL2-RA | Interleukin-2 receptor subunit alpha |  | 5.555 ± 0.37 |
| P24394 | IL-4RA | Interleukin-4 receptor subunit alpha |  | 3.532 ± 0.443 |
| P05231 | IL6 | Interleukin-6 |  | 4.806 ± 0.98 |
| P08887 | IL-6RA | Interleukin-6 receptor subunit alpha |  | 12.698 ± 0.404 |
| Q9UKP3 | ITGB1BP2 | Melusin | Y |  |
| P05107 | ITGB2 | Integrin beta-2 |  | 5.887 ± 0.402 |
| Q9Y624 | JAM-A | Junctional adhesion molecule A |  | 7.928 ± 0.396 |
| Q96D42 | KIM1 | Kidney Injury Molecule |  | 9.079 ± 1.61 |
| Q92876 | KLK6 | Kallikrein-6 |  | 5.459 ± 0.456 |
| P01130 | LDL receptor | Low-density lipoprotein receptor |  | 5.105 ± 0.912 |
| P41159 | LEP | Leptin |  | 7.706 ± 1.492 |
| P78380 | LOX-1 | Lectin-like oxidized LDL receptor 1 |  | 7.853 ± 0.615 |
| P06858 | LPL | Lipoprotein lipase |  | 11.15 ± 0.306 |
| P36941 | LTBR | Lymphotoxin-beta receptor |  | 7.307 ± 0.289 |
| Q9UEW3 | MARCO | Macrophage receptor MARCO |  | 7.711 ± 0.232 |
| P02144 | MB | Myoglobin |  | 10.621 ± 0.814 |
| P13500 | MCP-1 | Monocyte chemotactic protein 1 |  | 5.605 ± 0.46 |
| Q9NQ76 | MEPE | Matrix extracellular phosphoglycoprotein |  | 9.307 ± 0.529 |
| Q12866 | MERTK | Tyrosine-protein kinase Mer |  | 7.332 ± 0.434 |
| P39900 | MMP12 | Matrix metalloproteinase-12 |  | 9.17 ± 0.734 |
| P08253 | MMP-2 | Matrix metalloproteinase-2 |  | 4.734 ± 0.393 |
| P08254 | MMP-3 | Matrix metalloproteinase-3 |  | 10.389 ± 0.771 |
| P09237 | MMP7 | Matrix metalloproteinase-7 |  | 11.82 ± 0.179 |
| P14780 | MMP-9 | Matrix metalloproteinase-9 |  | 6.775 ± 0.802 |
| P05164 | MPO | Myeloperoxidase |  | 4.178 ± 0.514 |
| Q9Y6K9 | NEMO | NF-kappa-B essential modulator |  | 4.379 ± 0.668 |
| Q9UM47 | Notch 3 | Neurogenic locus notch homolog protein 3 |  | 6.711 ± 0.361 |
| NA | NT-proBNP | N-terminal prohormone brain natriuretic peptide |  | 8.942 ± 1.568 |
| O00300 | OPG | Osteoprotegerin |  | 5.392 ± 0.529 |
| P10451 | OPN | Osteopontin |  | 9.957 ± 0.457 |
| P05121 | PAI | Plasminogen activator inhibitor 1 |  | 7.27 ± 0.726 |
| Q13219 | PAPPA | Pappalysin-1 |  | 5.06 ± 0.649 |
| P25116 | PAR-1 | Proteinase-activated receptor 1 |  | 6.436 ± 0.489 |
| P09874 | PARP-1 | Poly [ADP-ribose] polymerase 1 |  | 2.444 ± 0.695 |
| Q8NBP7 | PCSK9 | Proprotein convertase subtilisin/kexin type 9 |  | 4.135 ± 0.689 |
| P04085 | PDGF subunit A | Platelet-derived growth factor subunit A |  | 5.905 ± 0.702 |
| P01127 | PDGF subunit B | Platelet-derived growth factor subunit B |  | 11.536 ± 0.636 |
| Q9BQ51 | PD-L2 | Programmed cell death 1 ligand 2 |  | 4.713 ± 0.323 |
| P16284 | PECAM-1 | Platelet endothelial cell adhesion molecule |  | 5.35 ± 0.323 |
| P49763 | PGF | Placenta growth factor |  | 12.108 ± 0.264 |
| O75594 | PGLYRP1 | Peptidoglycan recognition protein 1 |  | 9.753 ± 0.446 |
| P19957 | PI3 | Elafin |  | 6.371 ± 0.593 |
| P01833 | PIgR | Polymeric immunoglobulin receptor |  | 3.045 ± 0.198 |
| P98160 | PLC | Perlecan |  | 9.921 ± 0.09 |
| Q15166 | PON3 | Paraoxonase |  | 6.823 ± 0.593 |
| P51888 | PRELP | Prolargin |  | 8.773 ± 0.158 |
| Q9BQR3 | PRSS27 | Serine protease 27 |  | 10.999 ± 0.427 |
| Q16651 | PRSS8 | Prostasin |  | 9.919 ± 0.209 |
| P24158 | PRTN3 | Myeloblastin |  | 5.108 ± 0.703 |
| Q14242 | PSGL-1 | P-selectin glycoprotein ligand 1 |  | 4.395 ± 0.17 |
| P35247 | PSP-D | Pulmonary surfactant-associated protein D |  | 3.103 ± 0.93 |
| P26022 | PTX3 | Pentraxin-related protein PTX3 |  | 4.493 ± 0.471 |
| Q15109 | RAGE | Receptor for advanced glycosylation end products |  | 14.754 ± 0.111 |
| Q99969 | RARRES2 | Retinoic acid receptor responder protein 2 |  | 12.744 ± 0.235 |
| P00797 | REN | Renin |  | 7.388 ± 1.028 |
| Q9HD89 | RETN | Resistin |  | 8.92 ± 0.708 |
| P21583 | SCF | Stem cell factor |  | 10.667 ± 0.202 |
| Q96PL1 | SCGB3A2 | Secretoglobin family 3A member 2 |  | 5.29 ± 0.84 |
| P16581 | SELE | E-selectin |  | 12.66 ± 0.664 |
| P16109 | SELP | P-selectin |  | 10.67 ± 0.5 |
| Q8IW75 | SERPINA12 | Serpin A12 |  | 4.141 ± 1.381 |
| P78324 | SHPS-1 | Tyrosine-protein phosphatase non-receptor type substrate 1 |  | 5.095 ± 0.444 |
| Q9NQ25 | SLAMF7 | SLAM family member 7 |  | 5.979 ± 0.547 |
| P04179 | SOD2 | Superoxide dismutase [Mn], mitochondrial |  | 9.502 ± 0.119 |
| Q99523 | SORT1 | Sortilin |  | 9.541 ± 0.303 |
| Q9HCB6 | SPON1 | Spondin-1 |  | 3.584 ± 0.493 |
| Q9BUD6 | SPON2 | Spondin-2 |  | 9.845 ± 0.095 |
| P12931 | SRC | Proto-oncogene tyrosine-protein kinase Src |  | 5.567 ± 0.65 |
| Q01638 | ST2 | ST2 protein |  | 5.16 ± 0.663 |
| Q13043 | STK4 | Serine/threonine-protein kinase 4 |  | 2.603 ± 0.677 |
| P13726 | TF | Tissue factor |  | 7.486 ± 0.29 |
| Q07654 | TFF3 | Trefoil factor 3 |  | 9.816 ± 0.363 |
| P10646 | TFPI | Tissue factor pathway inhibitor |  | 10.304 ± 0.383 |
| P21980 | TGM2 | Protein-glutamine gamma-glutamyltransferase 2 |  | 7.818 ± 0.545 |
| P35442 | THBS2 | Thrombospondin-2 |  | 6.189 ± 0.2 |
| P40225 | THPO | Thrombopoietin |  | 4.026 ± 0.365 |
| Q02763 | TIE2 | Angiopoietin-1 receptor |  | 8.064 ± 0.258 |
| Q99727 | TIMP4 | Metalloproteinase inhibitor 4 |  | 5.013 ± 0.452 |
| Q5T2D2 | TLT-2 | Trem-like transcript 2 protein |  | 7.132 ± 0.407 |
| P07204 | TM | Thrombomodulin TM |  | 12.343 ± 0.211 |
| P19438 | TNF-R1 | Tumor necrosis factor receptor 1 |  | 11.176 ± 0.337 |
| P20333 | TNF-R2 | Tumor necrosis factor receptor 2 |  | 9.407 ± 0.367 |
| O00220 | TNFRSF10A | Tumor necrosis factor receptor superfamily member 10A |  | 6.56 ± 0.36 |
| O14798 | TNFRSF10C | Tumor necrosis factor receptor superfamily member 10C |  | 8.147 ± 0.373 |
| Q9Y6Q6 | TNFRSF11A | Tumor necrosis factor receptor superfamily member 11A |  | 10.54 ± 0.386 |
| O14836 | TNFRSF13B | Tumor necrosis factor receptor superfamily member 13B |  | 10.774 ± 0.552 |
| Q92956 | TNFRSF14 | Tumor necrosis factor receptor superfamily member 14 |  | 9.211 ± 0.375 |
| Q9Y275 | TNFSF13B | Tumor necrosis factor ligand superfamily member 13B |  | 7.685 ± 0.505 |
| P00750 | t-PA | Tissue-type plasminogen activator |  | 6.718 ± 0.793 |
| P02786 | TR | Transferrin receptor protein 1 |  | 5.976 ± 0.597 |
| O14763 | TRAIL-R2 | TNF-related apoptosis-inducing ligand receptor 2 |  | 9.123 ± 0.394 |
| P13686 | TR-AP | Tartrate-resistant acid phosphatase type 5 |  | 4.826 ± 0.457 |
| P00749 | uPA | Urokinase-type plasminogen activator |  | 5.579 ± 0.555 |
| Q03405 | U-PAR | Urokinase plasminogen activator surface receptor |  | 8.754 ± 0.501 |
| O43915 | VEGFD | Vascular endothelial growth factor D |  | 8.812 ± 0.297 |
| Q96IQ7 | VSIG2 | V-set and immunoglobulin domain-containing protein 2 |  | 7.647 ± 0.467 |
| P04275 | vWF | von Willebrand factor |  | 8.08 ± 0.688 |
| P47992 | XCL1 | Lymphotactin |  | 6.748 ± 0.493 |

**Supplementary Figure S1.** Chromatogram obtained using UV detection (λ = 400 nm) of nine standard SCFA mixture reacted with 2-nitrophenylhydrazides. Peak 1 = lactic acid, peak 2 = acetic acid, peak 3 = propionic acid, peak 4 = isobutyric acid, peak 5 = butyric acid, peak 6 = 2-methylbutyric acid, peak 7 = isovaleric acid, peak 8 = valeric acid, peak 9 = 2-ethylbutyric acid (IS), peak 10 = isocaproic acid.


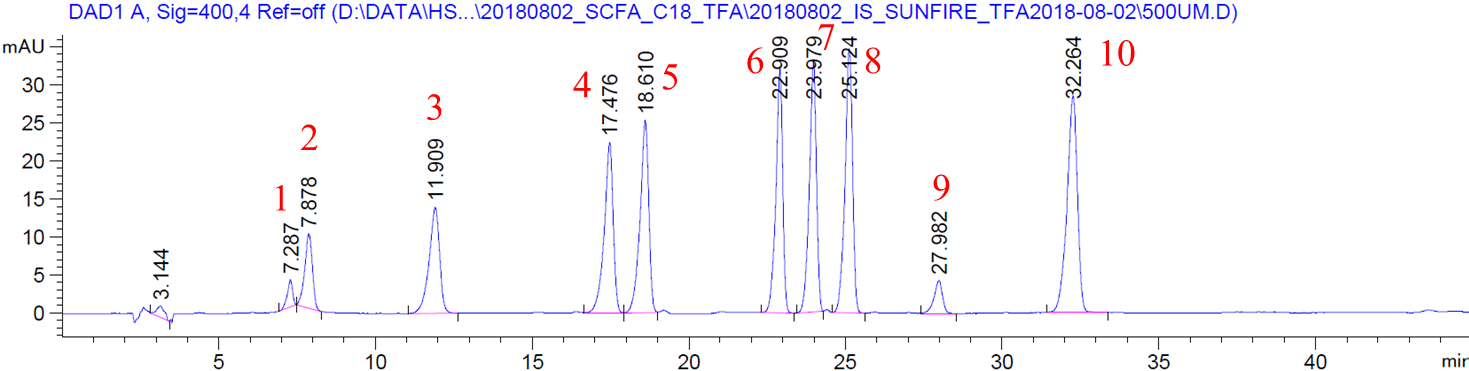


**Supplementary Figure S2**. Chromatogram of derivatized SCFA in the human serum sample. We compared the retention times with nine derivatization SCFA standards. The peak at retention time 7.27 min was annotated as lactic acid, retention time 25.13 min was annotated as valeric acid, and the retention time 27.98 min was the internal standard.


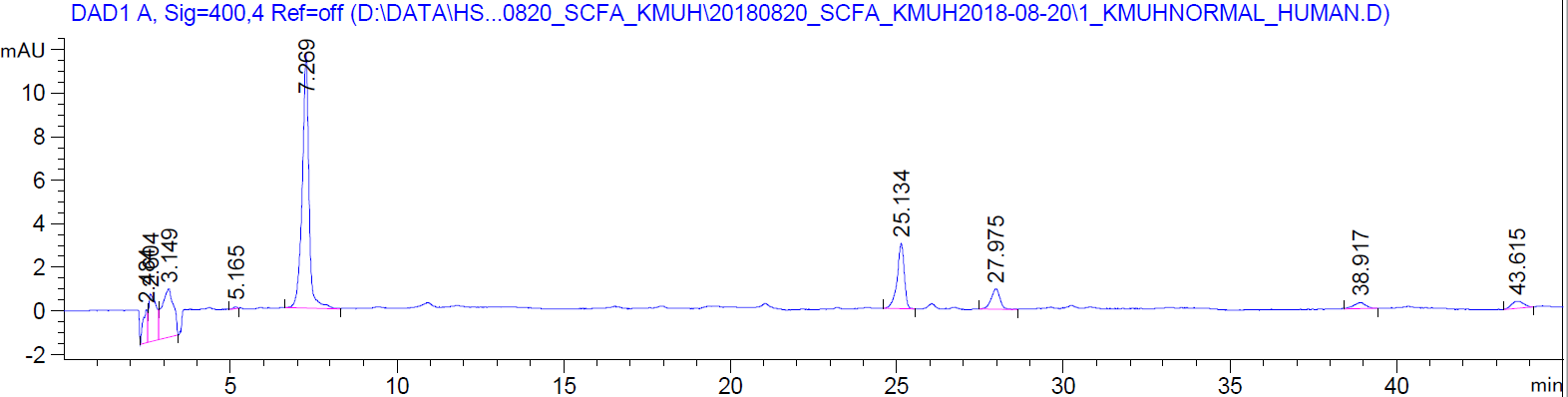


**Supplementary Figure S3.** Proposed causal diagram for the association between short-chain fatty acid and cardiovascular protein biomarkers


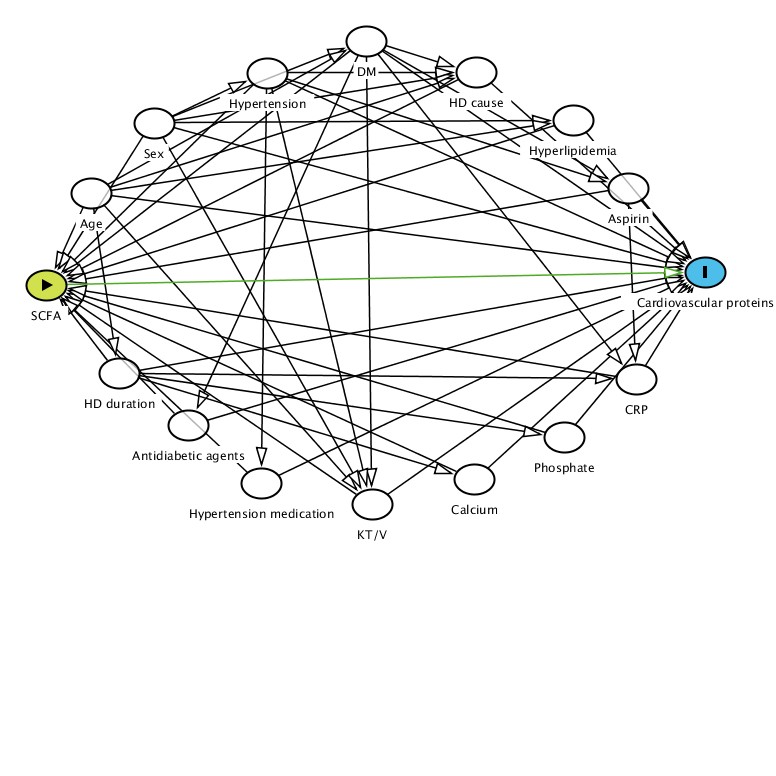


**Supplementary Figure S4.** Ranking proteins by *p*-value with bootstrapped confidence intervals around the ranks related to 2-methylbutyric acid


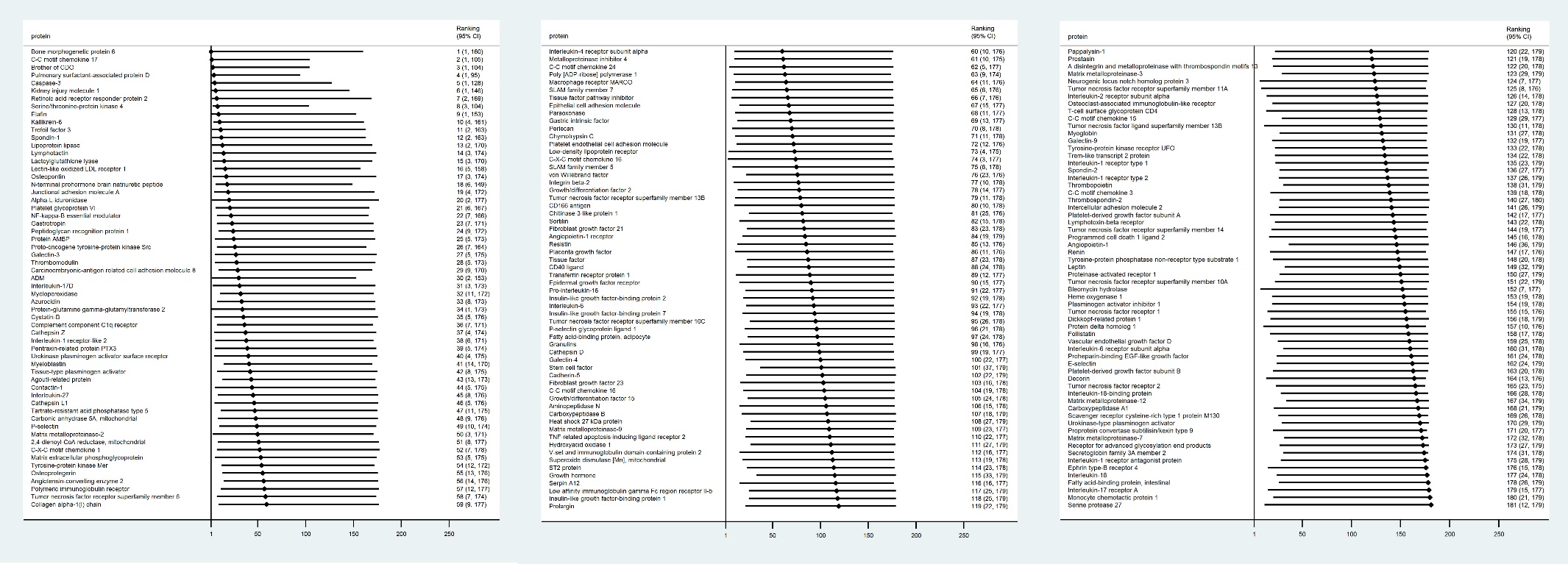


**Supplementary Table S2.** The β coefficient and 95% CI in linear regression models with age and sex adjustment between 2-methylbutyric acid and 181 cardiovascular proteins

| Cardiovascular protein name | β | 95% CI | *p* |
| --- | --- | --- | --- |
| Kidney injury molecule 1 | -1.54 | (-2.98 to -0.1) | 0.036 |
| C-C motif chemokine 17 | -1.34 | (-2.25 to -0.42) | 0.004 |
| Pulmonary surfactant-associated protein D | -1.15 | (-1.99 to -0.3) | 0.008 |
| N-terminal prohormone brain natriuretic peptide | -1.07 | (-2.36 to 0.21) | 0.101 |
| Bone morphogenetic protein 6 | -0.90 | (-1.33 to -0.47) | <0.001 |
| Carbonic anhydrase 5A, mitochondrial | -0.51 | (-1.43 to 0.42) | 0.284 |
| Agouti-related protein | -0.48 | (-1.31 to 0.34) | 0.251 |
| Alpha-L-iduronidase | -0.43 | (-0.98 to 0.11) | 0.119 |
| Lymphotactin | -0.39 | (-0.83 to 0.06) | 0.087 |
| Brother of CDO | -0.38 | (-0.64 to -0.11) | 0.006 |
| Osteopontin | -0.37 | (-0.81 to 0.07) | 0.097 |
| Fibroblast growth factor 23 | -0.37 | (-1.67 to 0.93) | 0.581 |
| Growth hormone | -0.36 | (-1.92 to 1.19) | 0.647 |
| Spondin-1 | -0.36 | (-0.74 to 0.02) | 0.067 |
| C-X-C motif chemokine 1 | -0.33 | (-0.94 to 0.28) | 0.290 |
| Hydroxyacid oxidase 1 | -0.32 | (-1.55 to 0.91) | 0.611 |
| Urokinase plasminogen activator surface receptor | -0.30 | (-0.76 to 0.16) | 0.201 |
| Interleukin-6 | -0.27 | (-1.14 to 0.59) | 0.538 |
| ADM | -0.24 | (-0.59 to 0.1) | 0.160 |
| Lipoprotein lipase | -0.24 | (-0.5 to 0.02) | 0.070 |
| Angiotensin-converting enzyme 2 | -0.23 | (-0.68 to 0.21) | 0.304 |
| Tartrate-resistant acid phosphatase type 5 | -0.23 | (-0.65 to 0.18) | 0.274 |
| Matrix extracellular phosphoglycoprotein | -0.23 | (-0.66 to 0.2) | 0.292 |
| Contactin-1 | -0.23 | (-0.62 to 0.17) | 0.261 |
| Matrix metalloproteinase-2 | -0.20 | (-0.56 to 0.17) | 0.289 |
| Interleukin-4 receptor subunit alpha | -0.19 | (-0.58 to 0.19) | 0.319 |
| Interleukin-27 | -0.19 | (-0.53 to 0.15) | 0.270 |
| Tyrosine-protein kinase Mer | -0.19 | (-0.55 to 0.17) | 0.294 |
| Low affinity immunoglobulin gamma Fc region receptor II-b | -0.19 | (-1.02 to 0.65) | 0.663 |
| SLAM family member 5 | -0.14 | (-0.48 to 0.2) | 0.409 |
| Leptin | -0.12 | (-1.34 to 1.1) | 0.846 |
| Pappalysin-1 | -0.12 | (-0.66 to 0.43) | 0.672 |
| Collagen alpha-1(I) chain | -0.11 | (-0.33 to 0.11) | 0.317 |
| V-set and immunoglobulin domain-containing protein 2 | -0.11 | (-0.54 to 0.32) | 0.612 |
| Renin | -0.10 | (-1.07 to 0.87) | 0.837 |
| CD166 antigen | -0.10 | (-0.35 to 0.15) | 0.447 |
| Polymeric immunoglobulin receptor | -0.10 | (-0.28 to 0.09) | 0.307 |
| Tissue factor | -0.09 | (-0.35 to 0.17) | 0.496 |
| TNF-related apoptosis-inducing ligand receptor 2 | -0.09 | (-0.43 to 0.25) | 0.610 |
| Aminopeptidase N | -0.09 | (-0.4 to 0.23) | 0.589 |
| Platelet-derived growth factor subunit A | -0.09 | (-0.72 to 0.55) | 0.792 |
| Tumor necrosis factor ligand superfamily member 13B | -0.08 | (-0.54 to 0.38) | 0.724 |
| C-C motif chemokine 3 | -0.08 | (-0.61 to 0.45) | 0.769 |
| Protein AMBP | -0.08 | (-0.18 to 0.03) | 0.152 |
| Tumor necrosis factor receptor superfamily member 11A | -0.07 | (-0.42 to 0.28) | 0.695 |
| Interleukin-2 receptor subunit alpha | -0.07 | (-0.41 to 0.27) | 0.695 |
| Neurogenic locus notch homolog protein 3 | -0.07 | (-0.39 to 0.26) | 0.690 |
| Trem-like transcript 2 protein | -0.06 | (-0.4 to 0.28) | 0.729 |
| Tyrosine-protein kinase receptor UFO | -0.06 | (-0.38 to 0.27) | 0.728 |
| Heat shock 27 kDa protein | -0.06 | (-0.27 to 0.16) | 0.597 |
| Interleukin-1 receptor type 1 | -0.06 | (-0.37 to 0.26) | 0.729 |
| P-selectin glycoprotein ligand 1 | -0.05 | (-0.21 to 0.12) | 0.565 |
| Tyrosine-protein phosphatase non-receptor type substrate 1 | -0.04 | (-0.45 to 0.37) | 0.837 |
| Prostasin | -0.04 | (-0.23 to 0.15) | 0.676 |
| E-selectin | -0.04 | (-0.63 to 0.56) | 0.905 |
| A disintegrin and metalloproteinase with thrombospondin motifs 13 | -0.04 | (-0.2 to 0.13) | 0.677 |
| Follistatin | -0.03 | (-0.52 to 0.45) | 0.889 |
| Bleomycin hydrolase | -0.03 | (-0.43 to 0.36) | 0.863 |
| Programmed cell death 1 ligand 2 | -0.03 | (-0.33 to 0.26) | 0.831 |
| Prolargin | -0.03 | (-0.17 to 0.11) | 0.669 |
| Platelet-derived growth factor subunit B | -0.03 | (-0.59 to 0.53) | 0.919 |
| Superoxide dismutase [Mn], mitochondrial | -0.03 | (-0.14 to 0.08) | 0.621 |
| Heme oxygenase 1 | -0.02 | (-0.31 to 0.26) | 0.868 |
| Vascular endothelial growth factor D | -0.02 | (-0.29 to 0.25) | 0.890 |
| Urokinase-type plasminogen activator | -0.02 | (-0.54 to 0.51) | 0.944 |
| Scavenger receptor cysteine-rich type 1 protein M130 | -0.02 | (-0.47 to 0.44) | 0.937 |
| Tumor necrosis factor receptor 2 | -0.02 | (-0.36 to 0.32) | 0.926 |
| Spondin-2 | -0.01 | (-0.1 to 0.07) | 0.744 |
| Interleukin-1 receptor antagonist protein | -0.01 | (-0.55 to 0.53) | 0.973 |
| Decorin | -0.01 | (-0.17 to 0.15) | 0.926 |
| Interleukin-18 | -0.01 | (-0.47 to 0.45) | 0.975 |
| Ephrin type-B receptor 4 | 0.00 | (-0.25 to 0.24) | 0.974 |
| Interleukin-17 receptor A | 0.00 | (-0.34 to 0.34) | 0.986 |
| Receptor for advanced glycosylation end products | 0.00 | (-0.1 to 0.1) | 0.959 |
| Serine protease 27 | 0.00 | (-0.39 to 0.39) | 0.999 |
| Monocyte chemotactic protein 1 | 0.00 | (-0.43 to 0.43) | 0.992 |
| Matrix metalloproteinase-7 | 0.00 | (-0.16 to 0.17) | 0.958 |
| Fatty acid-binding protein, intestinal | 0.01 | (-0.41 to 0.42) | 0.980 |
| Interleukin-18-binding protein | 0.01 | (-0.23 to 0.25) | 0.933 |
| Secretoglobin family 3A member 2 | 0.01 | (-0.75 to 0.78) | 0.972 |
| Proprotein convertase subtilisin/kexin type 9 | 0.02 | (-0.58 to 0.61) | 0.956 |
| Tumor necrosis factor receptor 1 | 0.02 | (-0.28 to 0.32) | 0.884 |
| Dickkopf-related protein 1 | 0.02 | (-0.29 to 0.33) | 0.885 |
| Interleukin-6 receptor subunit alpha | 0.02 | (-0.34 to 0.39) | 0.897 |
| Thrombospondin-2 | 0.02 | (-0.15 to 0.2) | 0.788 |
| Tumor necrosis factor receptor superfamily member 10A | 0.03 | (-0.27 to 0.32) | 0.860 |
| Matrix metalloproteinase-12 | 0.03 | (-0.61 to 0.67) | 0.934 |
| Carboxypeptidase A1 | 0.03 | (-0.65 to 0.71) | 0.935 |
| Lymphotoxin-beta receptor | 0.03 | (-0.22 to 0.28) | 0.800 |
| Osteoclast-associated immunoglobulin-like receptor | 0.04 | (-0.15 to 0.22) | 0.702 |
| Proteinase-activated receptor 1 | 0.04 | (-0.35 to 0.42) | 0.857 |
| Galectin-9 | 0.04 | (-0.16 to 0.23) | 0.725 |
| Perlecan | 0.04 | (-0.05 to 0.12) | 0.376 |
| Protein delta homolog 1 | 0.04 | (-0.51 to 0.58) | 0.889 |
| Tumor necrosis factor receptor superfamily member 14 | 0.04 | (-0.31 to 0.4) | 0.815 |
| Proheparin-binding EGF-like growth factor | 0.04 | (-0.62 to 0.71) | 0.898 |
| T-cell surface glycoprotein CD4 | 0.05 | (-0.2 to 0.3) | 0.704 |
| Intercellular adhesion molecule 2 | 0.05 | (-0.32 to 0.42) | 0.792 |
| Plasminogen activator inhibitor 1 | 0.05 | (-0.59 to 0.7) | 0.879 |
| Thrombopoietin | 0.05 | (-0.29 to 0.4) | 0.768 |
| Interleukin-1 receptor type 2 | 0.05 | (-0.28 to 0.39) | 0.747 |
| Stem cell factor | 0.05 | (-0.14 to 0.25) | 0.580 |
| Epidermal growth factor receptor | 0.07 | (-0.14 to 0.27) | 0.533 |
| Angiopoietin-1 | 0.07 | (-0.58 to 0.72) | 0.836 |
| Granulins | 0.08 | (-0.19 to 0.34) | 0.571 |
| Placenta growth factor | 0.08 | (-0.16 to 0.32) | 0.495 |
| Angiopoietin-1 receptor | 0.09 | (-0.16 to 0.33) | 0.488 |
| Insulin-like growth factor-binding protein 7 | 0.10 | (-0.22 to 0.42) | 0.543 |
| Cadherin-5 | 0.10 | (-0.25 to 0.45) | 0.581 |
| Sortilin | 0.10 | (-0.17 to 0.37) | 0.466 |
| Macrophage receptor MARCO | 0.10 | (-0.1 to 0.3) | 0.331 |
| Tumor necrosis factor receptor superfamily member 10C | 0.10 | (-0.24 to 0.44) | 0.555 |
| Pro-interleukin-16 | 0.11 | (-0.24 to 0.46) | 0.534 |
| Cathepsin D | 0.11 | (-0.28 to 0.51) | 0.577 |
| C-C motif chemokine 15 | 0.12 | (-0.5 to 0.73) | 0.713 |
| C-X-C motif chemokine 16 | 0.12 | (-0.16 to 0.39) | 0.405 |
| Complement component C1q receptor | 0.12 | (-0.06 to 0.3) | 0.185 |
| Myoglobin | 0.12 | (-0.55 to 0.79) | 0.725 |
| Matrix metalloproteinase-3 | 0.12 | (-0.46 to 0.71) | 0.685 |
| Galectin-4 | 0.12 | (-0.32 to 0.57) | 0.580 |
| Platelet endothelial cell adhesion molecule | 0.13 | (-0.17 to 0.43) | 0.393 |
| Thrombomodulin | 0.14 | (-0.05 to 0.33) | 0.155 |
| Insulin-like growth factor-binding protein 2 | 0.14 | (-0.31 to 0.59) | 0.538 |
| Growth/differentiation factor 2 | 0.14 | (-0.21 to 0.5) | 0.428 |
| Integrin beta-2 | 0.15 | (-0.21 to 0.5) | 0.422 |
| Growth/differentiation factor 15 | 0.15 | (-0.38 to 0.67) | 0.586 |
| ST2 protein | 0.15 | (-0.45 to 0.75) | 0.628 |
| Cathepsin L1 | 0.15 | (-0.12 to 0.42) | 0.270 |
| Tissue factor pathway inhibitor | 0.16 | (-0.18 to 0.5) | 0.351 |
| C-C motif chemokine 16 | 0.18 | (-0.45 to 0.81) | 0.583 |
| Transferrin receptor protein 1 | 0.18 | (-0.38 to 0.74) | 0.530 |
| Matrix metalloproteinase-9 | 0.19 | (-0.53 to 0.91) | 0.605 |
| Fatty acid-binding protein, adipocyte | 0.19 | (-0.47 to 0.84) | 0.571 |
| Carboxypeptidase B | 0.19 | (-0.5 to 0.88) | 0.591 |
| Cathepsin Z | 0.19 | (-0.1 to 0.49) | 0.196 |
| Tumor necrosis factor receptor superfamily member 13B | 0.20 | (-0.29 to 0.69) | 0.431 |
| Metalloproteinase inhibitor 4 | 0.20 | (-0.2 to 0.6) | 0.320 |
| Tumor necrosis factor receptor superfamily member 6 | 0.20 | (-0.19 to 0.6) | 0.312 |
| Retinoic acid receptor responder protein 2 | 0.21 | (0.01 to 0.41) | 0.037 |
| Galectin-3 | 0.22 | (-0.08 to 0.53) | 0.154 |
| SLAM family member 7 | 0.22 | (-0.23 to 0.68) | 0.339 |
| Resistin | 0.23 | (-0.42 to 0.87) | 0.491 |
| Osteoprotegerin | 0.23 | (-0.2 to 0.65) | 0.297 |
| Interleukin-17D | 0.24 | (-0.1 to 0.57) | 0.162 |
| Chymotrypsin C | 0.24 | (-0.31 to 0.79) | 0.386 |
| P-selectin | 0.25 | (-0.21 to 0.71) | 0.289 |
| Paraoxonase | 0.25 | (-0.28 to 0.78) | 0.359 |
| Interleukin-1 receptor-like 2 | 0.26 | (-0.14 to 0.65) | 0.200 |
| 2,4-dienoyl-CoA reductase, mitochondrial | 0.26 | (-0.22 to 0.73) | 0.290 |
| Chitinase-3-like protein 1 | 0.26 | (-0.42 to 0.94) | 0.456 |
| von Willebrand factor | 0.26 | (-0.36 to 0.88) | 0.410 |
| Cystatin-B | 0.27 | (-0.12 to 0.66) | 0.182 |
| Insulin-like growth factor-binding protein 1 | 0.28 | (-0.99 to 1.55) | 0.667 |
| Junctional adhesion molecule A | 0.28 | (-0.06 to 0.63) | 0.109 |
| Serpin A12 | 0.28 | (-0.96 to 1.53) | 0.653 |
| Pentraxin-related protein PTX3 | 0.29 | (-0.15 to 0.72) | 0.200 |
| Carcinoembryonic-antigen related cell adhesion molecule 8 | 0.30 | (-0.12 to 0.72) | 0.158 |
| Peptidoglycan recognition protein 1 | 0.30 | (-0.11 to 0.71) | 0.151 |
| C-C motif chemokine 24 | 0.30 | (-0.3 to 0.91) | 0.321 |
| Trefoil factor 3 | 0.31 | (-0.01 to 0.63) | 0.060 |
| Poly [ADP-ribose] polymerase 1 | 0.32 | (-0.31 to 0.94) | 0.321 |
| Myeloperoxidase | 0.32 | (-0.14 to 0.78) | 0.169 |
| Protein-glutamine gamma-glutamyltransferase 2 | 0.33 | (-0.15 to 0.81) | 0.176 |
| Platelet glycoprotein VI | 0.35 | (-0.09 to 0.78) | 0.119 |
| CD40 ligand | 0.37 | (-0.71 to 1.46) | 0.502 |
| Low-density lipoprotein receptor | 0.37 | (-0.5 to 1.24) | 0.403 |
| Myeloblastin | 0.39 | (-0.24 to 1.02) | 0.221 |
| Kallikrein-6 | 0.39 | (-0.01 to 0.8) | 0.056 |
| Lactoylglutathione lyase | 0.42 | (-0.06 to 0.91) | 0.089 |
| Gastric intrinsic factor | 0.43 | (-0.51 to 1.38) | 0.369 |
| Tissue-type plasminogen activator | 0.44 | (-0.28 to 1.16) | 0.230 |
| Fibroblast growth factor 21 | 0.45 | (-0.78 to 1.67) | 0.477 |
| NF-kappa-B essential modulator | 0.45 | (-0.14 to 1.03) | 0.136 |
| Proto-oncogene tyrosine-protein kinase Src | 0.46 | (-0.17 to 1.09) | 0.153 |
| Lectin-like oxidized LDL receptor 1 | 0.47 | (-0.07 to 1.01) | 0.090 |
| Epithelial cell adhesion molecule | 0.49 | (-0.55 to 1.53) | 0.354 |
| Elafin | 0.53 | (0 to 1.06) | 0.049 |
| Azurocidin | 0.56 | (-0.25 to 1.36) | 0.174 |
| Gastrotropin | 0.57 | (-0.19 to 1.32) | 0.143 |
| Serine/threonine-protein kinase 4 | 0.62 | (0.01 to 1.22) | 0.046 |
| Caspase-3 | 0.63 | (0.13 to 1.12) | 0.013 |
